# Efficacy of individualized education in patients with type 2 diabetes mellitus: A randomized clinical study protocol

**DOI:** 10.1097/MD.0000000000023625

**Published:** 2020-12-11

**Authors:** Li Huang, Hongyan Guo, Lihua Xiu, Haowen Wang

**Affiliations:** a Department of Gastroenterology; b Department of Cardiology, People's Hospital of Chengyang District, Qingdao, China.

**Keywords:** diabetes mellitus, individualized education, protocol

## Abstract

**Objective:**

To assess the effect of the program of individualized diabetes education on type 2 diabetes mellitus (DM) patients.

**Methods:**

This is a single-center randomized controlled trial that will be implemented from December 2020 to April 2021. The experiment was granted through the Research Ethics Committee of People's Hospital of Chengyang District (03982765). Patients are randomly assigned to the study group and control group with 50 cases in each group. Patients who meet the following criteria will be included in our study: patients diagnosed with type 2 DM based on the World Health Organization diagnostic criteria in 1999; patients who can take part in the follow-up researches after discharge; patients who can provide the written informed consent. And the exclusion criteria include: the known mental or psychological disorders, for instance, severe anxiety disorders or depression; severe comorbidities, e.g. liver dysfunction, kidney failure, stroke, and cancer; Uncontrolled diabetes complications, for instance, infection, acidosis, as well as peripheral vascular disease. The clinical examination shall be conducted during each follow-up period, and the laboratory examination is implemented as necessary in the process of each hospital visit. At the end of the 6-month study, each patient's blood pressure, waist circumference, body mass index, blood lipids, as well as fasting blood glucose are evaluated.

**Results:**

Table 1 reveals the comparison of biochemical results and clinical results between the control group and the study group.

**Conclusion:**

Individualized diabetes education may improve the clinical outcomes in patients with type 2 DM.

**Trial registration:**

The protocol was registered in Research Registry (researchregistry6232).

## 1 Introduction

Diabetes mellitus (DM) is a leading cause of mortality worldwide.^[1,2]^ It is a kind of debilitating and complex disease that, if not properly controlled, can lead to serious and substantial negative health consequences.^[3]^ On the basis of World Health Organization, the type 2 DM is caused by the body's ineffective use of insulin. Currently, the prevalence of type 2 DM is a major national and global health problem. Type 2 DM accounts for 90% of all the cases of diabetes, and the forecasts for the future seem grim, as the number of diabetics worldwide is expected to increase to 552 million, along with increased complications and health-care costs.^[4]^ Although the increased availability of guidelines of evidence-based treatment and anti-hypoglycemic medications, the percentage of type 2 DM patients who fail to meet their blood glucose targets is still on the rise.^[5,6]^ One of the main reasons is the delay of intensive treatment despite poor glycemic control, which is known as therapeutic or clinical inertia.^[7]^ An analysis of DM patients who adhered to diet therapy indicated that only 20 to 40% followed their diet guidelines.

Because of the complex etiology of long-term complications of type 2 DM and the existence of a range of changeable risk factors, multifactorial treatment is often required.^[8,9]^ There are several factors that affect compliance with prescribed treatment, containing knowledge levels, the environment, habits as well as attitudes.^[10,11]^ At present, most programs of diabetes education are knowledge-based, emphasizing self-care, lifestyle change, and the active participation in the management of disease.^[12,13]^ These educational programs are usually targeted at a group of patients. There is a lack of information about the impact of personality and individualized diabetes education on the outcomes of type 2 DM management. Hence, we carry out this randomized controlled research protocol to assess the effect of the program of individualized diabetes education on type 2 DM patients.

## 2 Methods

### 2.1 Study design

This is a randomized controlled and single-center study that will be implemented from December 2020 to April 2021. The experiment was granted through the Research Ethics Committee of People's Hospital of Chengyang District (03982765) and registered in Research Registry (researchregistry6232).

### 2.2 Patients and randomization

Patients are randomly assigned to the study group and control group with 50 cases in each group. Patients who meet the following criteria will be included in our study: patients diagnosed with type 2 DM based on the World Health Organization diagnostic criteria in 1999; patients who can take part in the follow-up researches after discharge; patients who can provide the written informed consent. And the exclusion criteria include: the known mental or psychological disorders, for instance, severe anxiety disorders or depression; severe comorbidities, e.g., liver dysfunction, kidney failure, stroke and cancer; uncontrolled diabetes complications, for instance, infection, acidosis, as well as peripheral vascular disease.

### 2.3 Interventions

Individualized education is provided to the study group patients by face-to-face counseling over 1 hour, based on the personality of the patient. The patient's knowledge on diabetes and self-care is assessed, and a tailored self-care plan is provided to each patient after the counseling. These plans are jointly developed by the nursing educators and a clinical psychologist in our hospital, covering dietary modification, exercises programs, adherence to medications, self-monitoring of blood glucose and blood pressure, and psychological counseling.

Education focuses on the significance of self-care programs and guiding patients to adhere to medications. A detailed explanation of the pathogenesis of diabetes, role of medications, and the use of self-monitoring equipment are provided to address patient's concerns and queries. The family members of patients are encouraged to help monitor the medication compliance. After discharge, all the patients are followed up in our outpatient department for 6 months at the end of each calendar month. All the patients are invited to participate in a 3-monthly forum held in our hospital's educational facilities. On the forum, patients are encouraged to exchange their ideas and views on the self-care of diabetes and share their progress in managing diabetes. In control group, patients receive face-to-face education for an hour in a group during their hospitalization. These educational courses are implemented via a same group of nurses who provided educational courses for study group. The main contents of education involve the basic knowledge of diabetes, the compliance and proper use of antidiabetic medications, the changes of lifestyle and diet, and the self-monitoring of the levels of blood glucose. Personality evaluation is not conducted and the plan of personalized self-care is not offered.

### 2.4 Assessment of outcomes

The clinical examination shall be conducted during each follow-up period, and the laboratory examination is implemented as necessary in the process of each hospital visits. At the end of the 6-month study, each patient's blood pressure, waist circumference, body mass index, blood lipids, as well as fasting blood glucose are evaluated.

### 2.5 Statistical analysis

The analysis of all data can be carried out with the software of IBM SPSS Statistics for Windows, version 20. Subsequently, all data can be represented with proper features, for example, median, mean, percentage, and the standard deviation. For each group, their corresponding qualitative parameters are assessed through the *t* test. And χ^2^ tests are applied for the determination of categorical variables. When *P* is less than .05, it is considered to be significant in statistics.

## 3 Results

Table 1 reveals the comparison of biochemical results and clinical results between the control group and the study group.

**Table 1:** The comparison of biochemical results and clinical results between the control group and the study group.

Parameter	Intervention group (n = 50)	Control group (n = 50)	*P* value
Waist circumference			
Body mass index			
Systolic blood pressure			
Diastolic blood pressure			
Fasting blood glucose			
Triglyceride			
Low density lipoprotein			
High-density lipoprotein			
Glycosylated hemoglobin			

## 4 Discussion

Some articles have suggested that the education of type 2 DM patients is related to the improvement of prognosis, which may be multifaceted.^[14–16]^ Individualized counseling or education, as well as a written plan of self-care on the basis of patient's personality, may improve patients’ motivation in adhering to dietary and life style modification measures.^[17]^ This is supported via significant reductions in waist circumference, body mass index, and body weight, which are known to help diabetics control blood pressure, cholesterol, and blood sugar.^[17]^ Moreover, a recent research suggested that the knowledge of diabetes was closely related to good control of blood glucose and medication adherence.^[18]^ Despite the patients’ medication compliance is not evaluated in our current study, self-care programs and individualized education may improve the patients’ compliance with cardiovascular and hypoglycemia medications, so as to better control blood lipids, blood pressure, and blood glucose. Although the psychologists are involved in the project, there are no psychosocial results as endpoints. This may be a weakness in research design and in-depth researches are needed.

## 5 Conclusion

Individualized diabetes education may improve the clinical outcomes in patients with type 2 DM.

## Author contributions

HW designs the protocol. LX reviews the protocol. HG performs the data collection. LH finishes the manuscript. All of the authors approved the submission.

**Conceptualization:** Lihua Xiu.

**Data curation:** Lihua Xiu.

**Funding acquisition:** Haowen Wang.

**Methodology:** Hongyan Guo.

**Project administration:** Hongyan Guo.

**Writing – original draft:** Li Huang.
